# A Novel High-Intensity Focused Ultrasound-Treated Herpes Simplex Virus 2 Vaccine Induces Long-Term Protective Immunity against Lethal Challenge in Mice

**DOI:** 10.1128/mSphere.00859-20

**Published:** 2020-12-23

**Authors:** Juhua Xiao, Xin Zhou, Ye Luo, Shuang Wang, Zhili Yang, Yingchun Yi, Hui Xiong

**Affiliations:** a Department of Ultrasound, Jiangxi Provincial Maternal and Child Health Hospital, Nanchang, China; b Department of Gynaecology, Jiangxi Provincial Maternal and Child Health Hospital, Nanchang, China; c Jiangxi Health Vocational College, Nanchang, China; Columbia University

**Keywords:** herpes simplex virus 2, high-intensity focused ultrasound, long-term immunity, vaccine efficacy

## Abstract

High-intensity focused ultrasound (HIFU), a noninvasive ablation therapy that has been widely used clinically in ablation of solid tumors, induces immune sensitization. We therefore in this study investigated whether HIFU treatment could enhance the efficacy of a herpes simplex virus 2 (HSV-2) vaccine. First, we observed that in HSV-2-positive cervical intraepithelial neoplasia (CIN) II patients, HIFU treatment induced significantly higher anti-HSV-2 neutralization response than surgical removal. Next, we tested the efficacy of HIFU-treated, UV-inactivated HSV-2-infected cells as a proof-of-concept vaccine in mice. Our data showed that HIFU-treated formulation significantly enhanced HSV-2 antibody titers and neutralization titers, compared to UV-, microwave (MW)-, or freeze-thaw (FT)-treated formulations. HIFU treatment also promoted the Th1/2 cell-mediated response. A long-term full protection was observed in mice that received the HIFU-treated formulation, and no weight loss was detected. Our findings indicate that the novel application of HIFU in vaccine production may represent a rational way to improve vaccine efficacy.

**IMPORTANCE** High-intensity focused ultrasound (HIFU) is mainly used in tumor ablation and tumor vaccinology study. It has been shown to induce immune sensitization and enhance tumor responsiveness to other therapies. Our study has shown enhanced anti-HSV-2 response in HIFU-treated CIN II patients. Furthermore, in a murine model, we have demonstrated that HIFU-treated HSV-2 vaccine induced long-term protective immunity against lethal challenge. Our findings indicate that the novel application of HIFU in vaccine production may represent a rational way to improve vaccine efficacy.

## Abstract (precis)

High-intensity focused ultrasound (HIFU) is mainly used in tumor ablation and tumor vaccinology study. It has been shown to induce immune sensitization and enhance tumor responsiveness to other therapies.

## INTRODUCTION

High-intensity focused ultrasound (HIFU) is a noninvasive therapy that uses an ultrasonic energy beam to treat malignant tissue (1). In recent years, HIFU has received increasing attention in the research and clinical applications of tumor treatment (2, 3). Solid tumors successfully treated by HIFU include pancreas, prostate, kidney, breast, and liver tumors, while the application of HIFU in treating uterine fibroids has also been approved by the FDA in the United States (4–7). HIFU causes tissue damage through two main mechanisms: heat and inertial cavitation. Irreversible cell death occurs when the temperature reaches above 56°C for at least 1 s (1). HIFU treatment can rapidly raise the temperature above 80°C at the focus and lead to effective cell killing (8, 9). Besides the thermal effect, HIFU treatment can also cause the formation of microbubbles, designated inertial cavitation, which rapidly collapse and result in cell necrosis (1). Consequently, HIFU induces effective cell death through a combination of thermal insult and mechanical stresses at a microscopic level.

In addition to the ablation of solid tumors, recent studies have revealed that HIFU can also modulate immune responses in HIFU-treated patients and animal models. Many cancer types including neuroblastoma are usually immunologically unresponsive, while a recent study on a murine model showed that HIFU treatment induced immune sensitization and enhanced tumor responsiveness to checkpoint inhibitor therapy (10). Our previous study also showed that among 152 cases of cervical intraepithelial neoplasia I (CIN I) combined with human papillomavirus (HPV) infection, HIFU treatment of CIN I also resulted in 89.47% of patients becoming HPV negative (11). These observations indicate that HIFU might present a novel immunoadjuvant modality to enhance immune response, which encouraged us to investigate whether HIFU could be used to enhance vaccine efficacy by using a proof-of-concept herpes simplex virus 2 (HSV-2) vaccine as a model.

HSV-2 is a sexually transmitted virus with no effective vaccine available, representing one of the main health concerns worldwide (12–14). HSV-2 has many glycoproteins on its surface and requires a few for successful infection; however, glycoprotein D (gD) is the one receiving much attention. gD is the protein that the virus utilizes to bind to receptor on the target cell surface and therefore is targeted by most neutralizing antibodies (15, 16).

**FIG 1:**
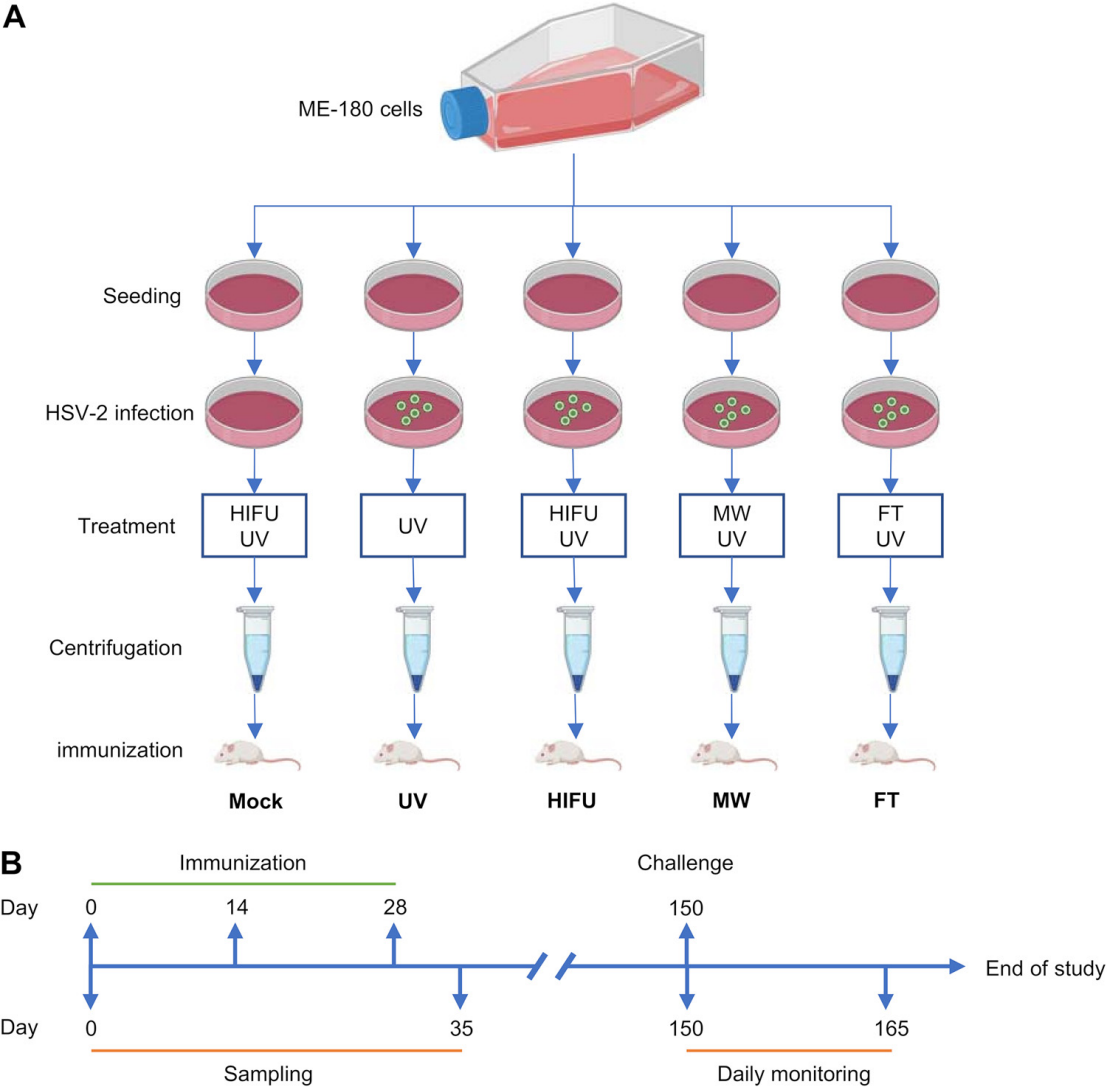
Schematic illustration for vaccine preparation and animal immunization schedule. (A) ME-180 cells preseeded in cell culture dishes were either mock infected or infected with HSV-2 at an MOI of 1, and then cells were washed and treated with HIFU, MW, or FT, followed by UV treatment. Treated cells were then centrifuged, and supernatants were harvested and used for injections. (B) Mice were injected intramuscularly with vaccine twice at 2-week intervals and then rested until day 150, when they were challenged intravaginally with a lethal dose of HSV-2. Blood and vaginal wash samples were harvested before the first injection and 1 week after the final injection. Weight and survival rate were monitored daily for 15 days.

In the current study, we first analyzed the HSV-2 neutralization titers of sera obtained from CIN II patients with HSV-2 infection before and after surgical or HIFU treatment, and our data revealed that HIFU significantly enhanced neutralizing antibody levels in sera. Subsequently, we tested the efficacy of HIFU-treated, UV-inactivated HSV-2-infected cells as a proof-of-concept vaccine in mice (Fig. 1). Our study showed that the HIFU-treated formulation significantly enhanced HSV-2-specific antibody and cell-mediated responses and that such enhancement provided long-term protection against lethal challenge. Our findings have revealed a novel application of HIFU in vaccine production.

## RESULTS

### HIFU treatment enhances anti-HSV-2 response in patients.

**FIG 2:**
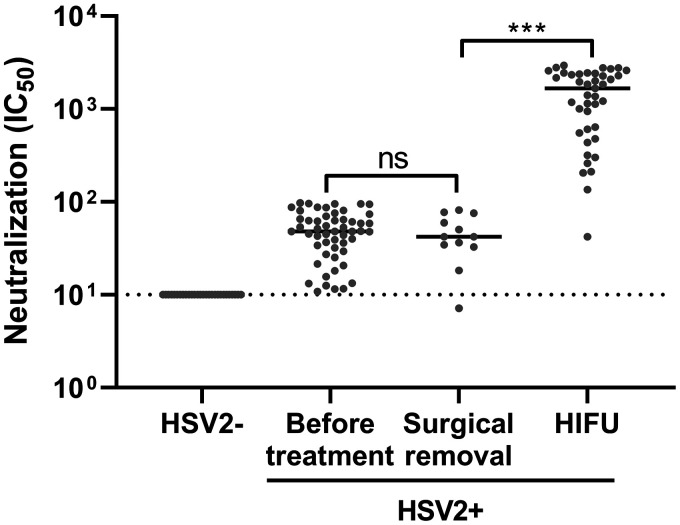
Serum neutralization activity against HSV-2 before and after HIFU treatment in CIN II patients. Blood samples were collected from CIN II patients on the day of diagnosis and 1 month after either surgical or HIFU treatment. Serum neutralization activity against HSV-2 was tested and calculated as IC_50_. ns, statistically not significant; ***, *P* < 0.001.

Serum samples were collected, both before and after treatment, from 81 CIN II patients who went through surgical or HIFU treatment and tested for HSV-2 neutralization. Of the 81 patients, 28 were HSV-2 negative while 53 were positive, as determined by HSV-2 neutralization assay. Among the 53 HSV-2-positive patients, 12 received surgical treatment while the remaining 41 received HIFU treatment for CIN II. Anti-HSV-2 neutralization assay showed that patients with surgical treatment had a neutralizing titer similar to that before treatment, whereas HIFU-treated patients showed significantly enhanced HSV-2 neutralization in sera compared to the pretreatment samples, indicating that HIFU treatment enhanced anti-HSV-2 immune response (Fig. 2).

### HIFU-treated vaccine increases HSV-2-specific antibody responses.

To investigate whether HIFU treatment could be used in vaccine preparation to enhance vaccine efficacy, we next used HIFU-treated HSV-2-infected cells as a proof-of-concept vaccine and tested its efficacy in immune induction activity. HIFU-treated uninfected cells and UV-, microwave (MW)-, and freeze-thaw (FT)-treated HSV-2-infected cells were used as controls (Fig. 1A). Mice were injected twice at 2-week intervals, and samples were taken before the first injection and 1 week after the final injection (Fig. 1B).

**FIG 3:**
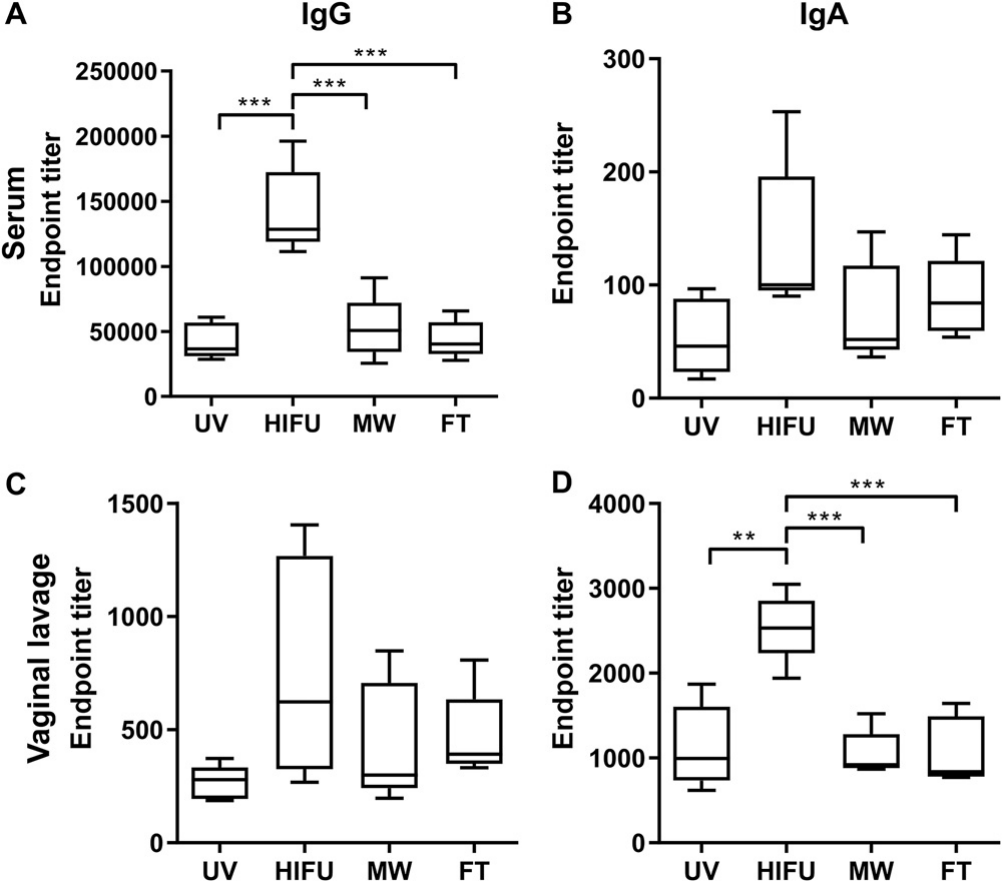
HSV-2 gD-specific IgG and IgA titers in serum and vaginal lavage samples of immunized mice. One week after the final immunization, blood and vaginal wash samples were collected and HSV-2 gD-specific IgG and IgA in serum and vaginal lavage samples were determined by endpoint ELISA. Data shown are mean ± SD from *n* = 5 for each group. **, *P* < 0.01; ***, *P* < 0.001.

HSV-2 gD-specific IgG and IgA titers in sera and vaginal lavage samples were first measured by enzyme-linked immunosorbent assay (ELISA). As shown in Fig. 3, HIFU formulation significantly increased gD-specific IgG in sera and IgA in vaginal lavage samples, compared to other control groups. An enhancement in serum IgA and vaginal IgG, albeit not statistically significant, was also observed in mice receiving HIFU-treated vaccine. In contrast, UV-, MW-, and FT-treated vaccine did not show an apparent difference in gD-specific antibodies in these two sample types.

### HIFU-treated vaccine enhances neutralization activity in sera and vaginal lavage samples.

**FIG 4:**
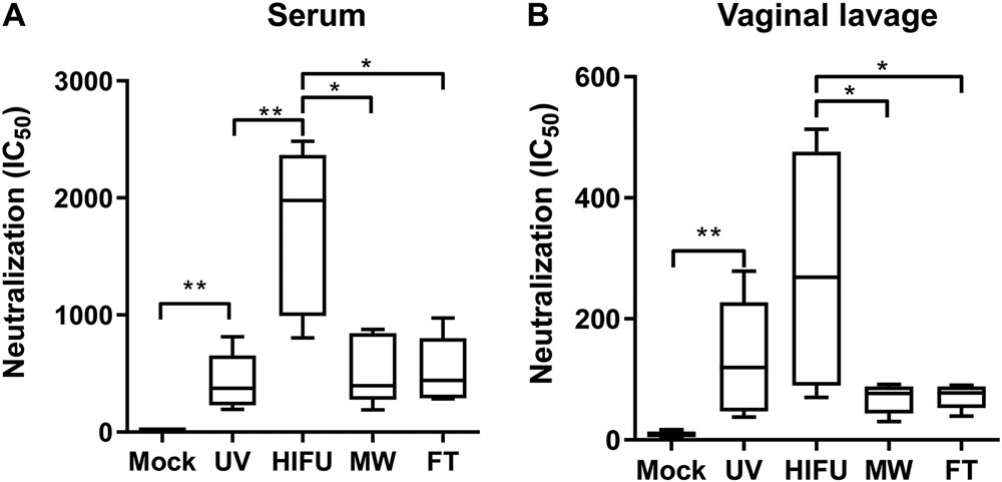
HSV-2 neutralization titer of sera and vaginal lavage samples from immunized mice. One week after the final immunization, blood and vaginal wash samples were collected, sera (A) and vaginal lavage samples (B) were serially diluted, and their neutralization activity against HSV-2 was measured. IC_50_ was calculated for each sample. Data shown are mean ± SD from *n* = 5 for each group. *, *P* < 0.05; **, *P* < 0.01.

To test whether enhanced antibody response could also lead to enhancement in neutralization, *in vitro* neutralization against HSV-2 was performed. Compared to mock-treated mice, all vaccinated mice showed neutralization in both serum and vaginal lavage (Fig. 4A and B). Similar to ELISA results, HIFU-treated vaccine induced significantly enhanced neutralization titers in both sera and vaginal lavage samples. UV-treated vaccine also induced a noticeable level of increase in neutralization in vaginal samples, although it did not reach statistical significance compared to MW and FT formulations (Fig. 4A and B). Taken together, the data indicate that HIFU-treated vaccine could induce enhanced neutralizing immunity in both sera and vaginal mucosal sites.

### HIFU-treated vaccine induces balanced Th1/Th2 cell-mediated responses.

**FIG 5:**
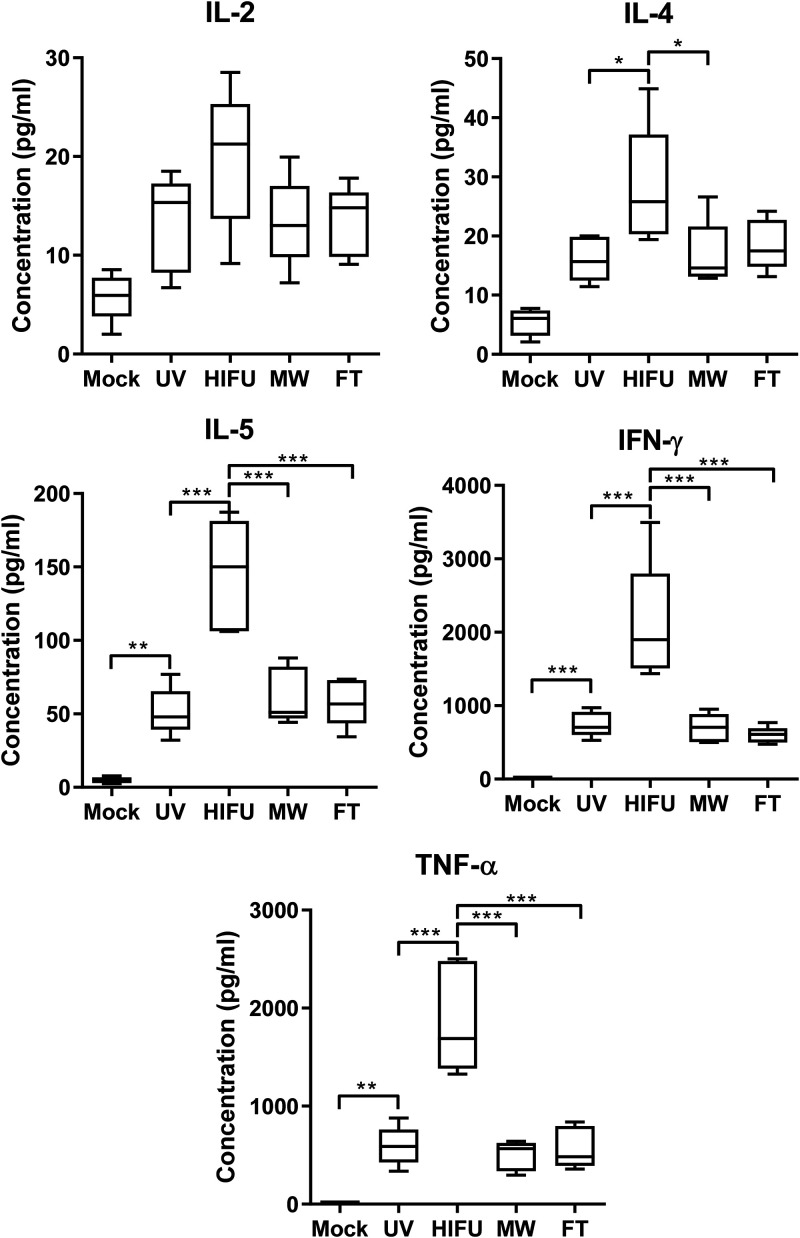
HSV-2 gD-specific Th1/Th2-related cytokine levels in restimulated mouse splenocytes. One week after the final immunization, mice were sacrificed, spleens were harvested, and splenocytes were isolated and restimulated with purified gD for 3 days. Th1/2-associated cytokines (IL-2, IL-4, IL-5, IFN-γ, and TNF) in the cell culture medium were then quantified with the Cytometric Bead Array mouse Th1/Th2 cytokine kit. Data shown are mean ± SD from *n* = 5 for each group. *, *P* < 0.05; **, *P* < 0.01; ***, *P* < 0.001.

Th1 and Th2 cell-mediated responses were also determined by quantification of Th1 (interleukin-2 [IL-2], gamma interferon [IFN-γ], and tumor necrosis factor [TNF])- and Th2 (IL-4 and IL-5)-associated cytokines in HSV-2 gD-restimulated splenocytes. As shown in Fig. 5, all vaccinated mice showed elevated Th1/Th2 cytokines, compared to mock-treated mice. Of the vaccinated mice, mice receiving HIFU-treated vaccine had significantly enhanced IL-4, IL-5, IFN-γ, and TNF levels. An increase in IL-2 was also observed in the HIFU-formulated vaccine group, although it did not reach statistical significance. No apparent difference was seen in the cytokine production in other vaccinated control groups. Taken together, the data indicate that HIFU-treated vaccine induces a balanced Th1/Th2 cell-mediated immune response.

### HIFU-treated vaccine induces long-term sterilizing protective immunity against lethal challenge.

**FIG 6:**
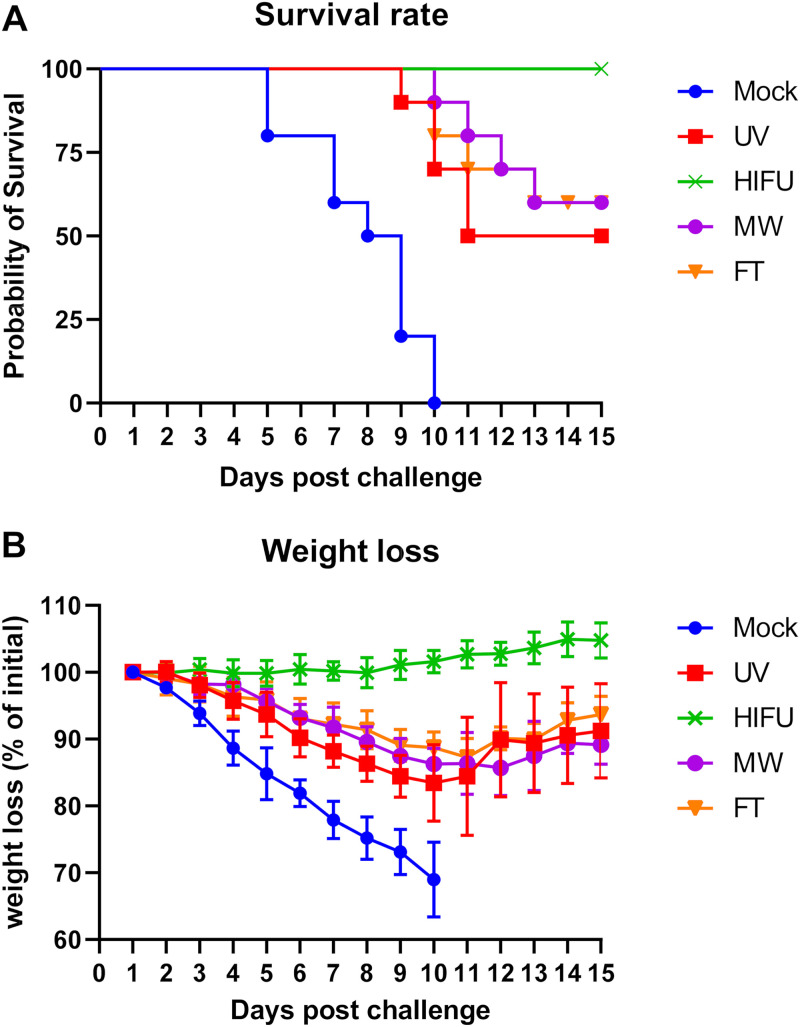
Survival rate and weight loss of vaccinated mice after lethal challenge. Mice were immunized twice with different vaccine formulations, rested for 115 days, and then challenged intravaginally with a lethal dose of HSV-2. Animal survival rate (A) and weight loss (B) were monitored daily for 15 days. Data shown are mean ± SD from *n* = 5 for each group.

To test whether a long-term protective immunity could be achieved by our HIFU-treated vaccine, mice were immunized twice as described above, then rested for 115 days, and then challenged intravaginally with lethal doses of HSV-2. After challenge, mouse survival and weight change were recorded on a daily basis (Fig. 1B). The mock-treated group showed a rapid pace of animal deaths and sharp weight loss, and all animals were dead on day 10 after challenge (Fig. 6A and B). Partial protection was observed in UV-, MW-, and FT-treated vaccine groups, with a survival rate of 60% in UV and MW groups and 50% in the FT group. All the three control vaccinated groups showed very similar trends in animal weight change. In detail, animals exhibited a steady weight loss at a pace slower than the mock-treated mice from day 1 to 10, and thereafter, animals started to slightly gain weight. HIFU-treated vaccine, in contrast, showed full protection against lethal viral challenge, as evidenced by the 100% survival rate after 15 days of monitoring as well as a slow but steady weight increase over the same time period (Fig. 6A and B). These data indicate that HIFU-treated HSV-2 vaccine induces long-term sterilizing protective immunity in mice.

### Conclusion.

As a proof-of-concept study, HIFU-treated vaccine enhances HSV-2-specific immune response in mice and the enhanced response can provide long-term protection against lethal challenge. Our findings reveal a novel application of HIFU in vaccine production.

## DISCUSSION

HIFU is a noninvasive ablation therapy to remove malignant tissues like solid tumors. Recently, studies have shown that HIFU can induce immune sensitization and enhance tumor responsiveness to immunological therapy and also successfully treat HPV infection, indicating that HIFU has an immune modulation effect (10, 11, 17). However, whether HIFU could be applied to vaccine preparation to produce more efficacious vaccines has not yet been investigated. To the best of our knowledge, this is the first study that has proved that an HIFU-treated HSV-2 vaccine has induced enhanced systemic and mucosal antibody responses and Th1/Th2 cellular responses. Such immune enhancement has generated long-term protection against lethal viral challenge. Our findings in the current study have provided a novel but rational application of HIFU in vaccine production.

Although we and others have demonstrated the immunomodulation effect of HIFU, the underlying mechanism still remains elusive. Although the mechanism remains unclear, HIFU has also been reported to trigger immune sensitization in solid tumors (10). HIFU usually uses the ultrasound frequency of 0.8 to 3.5 MHz, which carries high energy and delivers to a focused point (1, 18). HIFU has two main effects: heat and cavitation. HIFU can rapidly increase the temperature above 80°C at the focus (19). Microwave treatment can mimic the heat released by HIFU, but microwave-treated vaccine did not show a similar level of immune enhancement, indicating that HIFU-carried heat, at least not alone, is not the mechanism underlying enhancement of immune response by HIFU-treated vaccine. Another main effect of HIFU is induction of cavitation, which draws gas from solution to form microbubbles inside the cells. The rapid formation and collapse of these microbubbles can lead to cell burst and even breakup of big molecules in a unique way (20). We have included a group with freeze-thaw-treated vaccine, because freeze-thaw cycles, albeit in a different way, can also cause cell burst. Our data have shown that freeze-thaw-treated vaccine did not induce a similar response as the HIFU-treated version. Therefore, we speculate that HIFU treatment can break up immunogens to a level that will facilitate the efficient uptake and processing of these immunogens by antigen-presenting cells (APCs), leading to enhanced immune response. Further investigations are needed to understand the exact mechanism underlying the effect of HIFU on immune enhancement by vaccines.

Humoral and cellular responses are the two arms of adaptive response, which are interlinked, and consequently one relies on the other to function effectively (21). For example, antibody response needs the help from CD4^+^ T cells for isotype switch and in affinity maturation (22). To effectively activate T cells, the antigen needs to be processed into short peptides and presented by major histocompatibility complex class II (MHC II) (23). Therefore, efficient antigen processing is also one of the important steps to generate a good immune response. Although further investigations are required, it is possible that HIFU treatment can effectively break the viral antigens into small peptides, which can be recognized and presented by MHC II more efficiently. This theory is backed up by our measurement of cell-mediated responses in the current study. Our data have shown that, compared to UV-, MW-, and FT-treated vaccines, the HIFU-treated version has significantly enhanced Th1 and Th2 T cell responses.

To induce long-term protective immunity is a desirable goal for a vaccine. Usually, long-term immunity is established due to the long-living antigen-specific immune cells called memory cells (24). These memory immune cells, including memory B cells and T cells, persist in a resting state in the system and can be reactivated upon reexposure to the same antigen, and then they will rapidly propagate and produce immune effectors to prevent infection (25, 26). Our data in the current study have shown that HIFU-treated vaccine can fully protect mice from lethal HSV-2 challenge 115 days after final immunization. In contrast, other groups with control vaccine injections have had only partial protection. As 9 mouse days are equal to 1 human year, this means that HIFU-treated vaccine has induced protective immunity for about 13 human years (27).

As a proof-of-concept study, we in the current study used HSV-2-infected cells instead of purified virus as the source of immunogens for two main reasons. First, our initial investigation on HIFU-treated HSV-2-positive patients showed that a significant elevation of anti-HSV-2 immune response was detected after treatment. We believe that the products generated by HIFU treatment in HSV-2-positive cells in patients have played an important role in such immune enhancement. To better mimic this, we have decided to use HSV-2-infected cells as our vaccine candidate. Second, we have extensive experience with HIFU treatment of cells but not virus. Therefore, we lack the knowledge of appropriate HIFU parameters that should be used to treat virus. In addition, it remains unknown whether HIFU can induce cavitation in virus just like in cells. However, future studies should check whether HIFU-treated virus would generate similar immune responses as HIFU-treated virus-infected cells.

Taken together, our current study has shown a novel application of HIFU in vaccine production. Although further studies are required to explore the underlying mechanism and to confirm whether such findings can be transferrable to other animal models and humans, the adoption of HIFU can enhance vaccine-induced humoral and cellular immune responses and can offer long-term protective immunity in mice.

## MATERIALS AND METHODS

### Ethical statement.

All protocols involving human samples were reviewed and approved by the Ethics Review Committee of Jiangxi Provincial Maternal and Child Health Hospital and performed in accordance with the Declaration of Helsinki. Written consents were obtained from all participated subjects. All protocols involving animals were reviewed and approved by the Ethics Review Committee of Jiangxi Provincial Maternal and Child Health Hospital and performed in accordance with the provincial guidance on animal research.

### Patients and sample collection.

A total number of 81 patients diagnosed as CIN II from August 2018 to July 2019 were included in the study. Patients were treated by either surgical removal or HIFU. Serum samples were collected on the day of diagnosis and 1 month after treatment and used for HSV-2 neutralization.

### Cells and virus.

Human cervical epithelial cell line ME-180 and African green monkey kidney cell line Vero were purchased from the American Type Culture Collection (ATCC, Manassas, VA, USA) and cultured in Dulbecco’s modified Eagle medium (DMEM) (Sigma-Aldrich, Merck, Shanghai, China) supplemented with 10% fetal bovine serum (FBS) (Gibco, Thermo Fisher Scientific, Brazil) and penicillin-streptomycin (Sigma-Aldrich, Merck, Shanghai, China).

HSV-2 (strain 333) was also purchased from ATCC (Manassas, VA, USA) and propagated and titrated as previously described (28).

### Vaccine preparation.

The vaccine preparation protocol was illustrated in Fig. 1A. ME-180 cells were first seeded in 60-mm petri dishes and then mock infected or infected with HSV-2 (multiplicity of infection [MOI] of 1) for 24 h. Following infection, culture medium was removed, and cells were washed with phosphate-buffered saline (PBS) and resupplied with PBS. After washing, cells were then either mock treated or treated with HIFU (3.75-MHz probe scanning the cell monolayer at 5 to 10 mm/s for 3 times; CZF-1; Chongqing Haifu Technology Co. Ltd., Chongqing, China), microwave (MW) (700 W, 30 s for 3 times with 30-s intervals; NJL07-3; Nanjing Jiequan Microwave Development Co. Ltd., Nanjing, China), or freeze-thaw (FT) (3 cycles of −80°C, 10 min, and 37°C, 10 min) cycles, followed by UV treatment for all conditions to inactivate HSV-2. Treated cells were then resuspended and centrifuged at 10,000 × *g* for 10 min at 4°C. Supernatants were harvested, filtrated through a 0.22-μm membrane (Merck Millipore, Burlington, MA, USA), and used as proof-of-concept vaccines in the current study. Total protein in the supernatants was quantified by the Bradford protein quantification assay using the Pierce Coomassie (Bradford) protein assay kit (Thermo Fisher Scientific, Waltham, MA, USA), according to the manufacturer’s instructions. After quantification, vaccine was aliquoted and stored at −80°C.

### Mouse immunization, sample collection, and challenge.

Specific-pathogen-free (SPF) 6- to 8-week-old female BALB/c mice (Beijing Huabukang Co. Ltd., Beijing, China) were injected intramuscularly with 10-μg vaccine formulations twice at 2-week intervals. Blood and vaginal samples were taken before the first injection and 1 week after the final injection. For splenocyte restimulation *in vitro*, mice were sacrificed 1 week after the final injection and spleens were harvested. For lethal challenge, mice were rested for 115 days and challenged intravaginally with a lethal dose (200 50% lethal doses [LD_50_], 1 × 10^6^ PFU) of HSV-2, and mouse survival and weight loss were monitored daily. A detailed schedule is shown in Fig. 1B.

### HSV-2 gD-specific binding antibody titer quantification.

HSV-2 gD-specific binding antibody titer was determined by ELISA, as previously described with modifications (28). In brief, ELISA plates (Nunc MaxiSorp; Thermo Fisher Scientific, Shanghai, China) were coated with purified gD (1 μg/ml, 100 μl) overnight at 4°C. The next day, coated plates were washed with PBS with Tween 20 (PBST), blocked with 1% bovine serum albumin (BSA) (Sigma-Aldrich; Merck, Shanghai, China), and sequentially incubated with serially diluted samples and horseradish peroxidase (HRP)-conjugated goat anti-mouse IgG or IgA secondary antibodies (Protein Tech, Wuhan, China). After extensive washes, tetramethylbenzidine (TMB) substrate (Sigma-Aldrich, Merck, Shanghai, China) was added and incubated for 5 min in the dark. After the addition of stop solution, the absorbance of the plates was read at a testing wavelength of 450 nm and a reference wavelength of 800 nm using an ELISA plate reader (Tecan Sunrise; Tecan, Männedorf, Switzerland). Endpoint titer was calculated with the cutoff value of mean of negative control (samples from naive mice) plus 2 times standard deviation (SD).

### HSV-2 neutralization assay.

The HSV-2 neutralization titers of sera from CIN II patients as well as sera and vaginal lavage samples from immunized mice were tested by plaque assay, as previously described with modifications (28). In brief, samples were first heat inactivated for 30 min at 56°C and then serially diluted and incubated with 50 PFU of HSV-2 for 1 h at 37°C. Following incubation, the mixture was then added onto a Vero cell monolayer and cultured for 48 h. Then medium was removed, and cells were stained with crystal violet (Sigma-Aldrich, Merck, Shanghai, China). Following staining, cells were washed with PBS and plaques were counted. The neutralization was calculated as 50% inhibitory concentration (IC_50_), which represented the reciprocal of the sample dilution resulting in 50% virus inhibition.

### Th1/2-associated cytokine measurement.

One week after final injection, mice were sacrificed, spleens were harvested, and splenocytes were isolated using Lymphoprep (Stemcell, Vancouver, Canada). Freshly isolated splenocytes were then restimulated *in vitro* with purified HSV-2 gD for 3 days. After stimulation, cell culture medium was harvested and filtrated through a 0.45-μm membrane (Merck Millipore, Burlington, MA, USA). Th1/2-associated cytokines (IL-2, IL-4, IL-5, IFN-γ, and TNF) in the cell culture medium were then quantified with the Cytometric Bead Array mouse Th1/Th2 cytokine kit (BD Biosciences, San Diego, CA, USA), according to the manufacturer’s instructions.

### Statistical analysis.

All statistical analyses were done with GraphPad Prism 8.0 (GraphPad Software, Inc., San Diego, CA, USA). The Mann-Whitney test was used for comparison between groups, and the Kruskal-Wallis test followed by Dunn’s multiple-comparison test was used for comparisons among three or more groups. For all analyses, a *P* value less than 0.05 was considered statistically significant.
